# Sedative load of medications prescribed for older people with dementia in care homes

**DOI:** 10.1186/1471-2318-11-56

**Published:** 2011-09-30

**Authors:** Carole Parsons, Jane Haydock, Elspeth Mathie, Natasha Baron, Ina Machen, Elizabeth Stevenson, Sarah Amador, Claire Goodman

**Affiliations:** 1 School of Pharmacy, Queen's University Belfast, 97 Lisburn Road, Belfast, BT9 7BL, Northern Ireland, UK; 2 Centre for Research in Primary and Community Care, University of Hertfordshire, Hatfield, Hertfordshire, AL10 9AB, UK; 3 General Practice & Primary Care Research Unit, Institute of Public Health, Robinson Way, Cambridge, CB2 0SR, UK

## Abstract

**Background:**

The objective of this study was to determine the sedative load and use of sedative and psychotropic medications among older people with dementia living in (residential) care homes.

**Methods:**

Medication data were collected at baseline and at two further time-points for eligible residents of six care homes participating in the EVIDEM-End Of Life (EOL) study for whom medication administration records were available. Regular medications were classified using the Anatomical Therapeutic Chemical classification system and individual sedative loads were calculated using a previously published model.

**Results:**

At baseline, medication administration records were reviewed for 115 residents; medication records were reviewed for 112 and 105 residents at time-points 2 and 3 respectively. Approximately one-third of residents were not taking any medications with sedative properties at each time-point, while a significant proportion of residents had a low sedative load score of 1 or 2 (54.8%, 59.0% and 57.1% at baseline and time-points 2 and 3 respectively). More than 10% of residents had a high sedative load score (≥ 3) at baseline (12.2%), and this increased to 14.3% at time-points 2 and 3. Approximately two-thirds of residents (66.9%) regularly used one or more psychotropic medication(s). Antidepressants, predominantly selective serotonin re-uptake inhibitors (SSRIs), were most frequently used, while antipsychotics, hypnotics and anxiolytics were less routinely administered. The prevalence of antipsychotic use among residents was 19.0%, lower than has been previously reported for nursing home residents. Throughout the duration of the study, administration of medications recognised as having prominent sedative adverse effects and/or containing sedative components outweighed the regular use of primary sedatives.

**Conclusions:**

Sedative load scores were similar throughout the study period for residents with dementia in each of the care homes. Scores were lower than previously reported in studies conducted in long-term care wards which have on-site clinical support. Nevertheless, strategies to optimise drug therapy for care home residents with dementia which rely on clinicians external to the care home for support and medication review are required.

## Background

In the United Kingdom (UK), care homes are the main providers of long-term care for older people. They include care homes which provide 24-hour nursing care (nursing homes), those that provide personal care only (residential homes), and those with mixed provision, which offer both residential and nursing care [1]. Residential homes, which provide the majority of long-term care for older people in England, do not have on-site nursing provision, and rely on primary healthcare professionals, namely general practitioners (GPs), nurses and community pharmacists, for meeting healthcare needs, including prescribing and supply of medications and medication review [2].

Prescribing is one of the most common medical interventions experienced by older people resident in care homes [3,4]. Due to multiple medical conditions and polypharmacy (defined as the use of multiple medications and/or administration of more medications than are clinically indicated [5]), in addition to age-related changes in pharmacokinetics and pharmacodynamics, care home residents are at high risk of adverse drug events (ADEs) [6-8]. Older people with cognitive impairment are particularly susceptible to ADEs associated with sedative and psychotropic drugs [9]. The over-use of psychotropic drugs (antipsychotics, antidepressants, hypnotics, and anxiolytics) has been a particular concern in the literature on prescribing and use of medicines in care homes [10-17]; however, nursing homes have been the primary focus and these studies have not been limited to residents with cognitive impairment. Furthermore previous studies which compare psychotropic drug use among patients with and without dementia have been limited to specific classes of psychotropic drugs [18-21]. Recent interest has focused on the development of a measure to quantify residents' overall drug load [22-32]. Sedative drugs may include medications prescribed for intentional sedation, medications with which sedation is a prominent side-effect, or medications which yield sedation as a potential ADE [33,34]. A model has been developed to quantify the cumulative effect of taking multiple drugs with sedative properties, termed the sedative load [35,36]. This model has been utilised thus far to examine the sedative load among residents of long-term care wards in Finland [33,34]. An alternative model, the Drug Burden Index (a measure of a person's exposure to anticholinergic and sedative medications), has been used to examine exposure of residents of residential aged-care facilities in Australia to medications with anticholinergic and sedative properties [27,37,38]. However, to the best of the authors' knowledge, no studies have been conducted to date which use the sedative load model to examine prescribing of sedative medications in residents of residential homes. The objective of this study was to determine the sedative load and use of sedative and psychotropic medications among older people with dementia living in residential care homes.

## Methods

The eligible study population comprised all residents from six residential care homes participating in the EVIDEM-End Of Life (EOL) study for whom medication administration records were available at baseline and at the two further time-points (approximately sixteen weeks apart) at which data were collected over the 12-month data collection period. The EVIDEM-EOL study had a prospective design, tracking the events and care experienced by older people with dementia over two years, and was undertaken between April 2008 and June 2010. Ethical approval for the EVIDEM-EOL study was granted by the NHS Southampton and South West Hampshire Research Ethics Committee in July 2008 (MREC Ref: 08/H0502/74). All six care homes were registered to provide dementia care. Care home residents who were 65 years of age or older, and who had a documented diagnosis of dementia, or who were determined by senior care home staff as having cognitive impairment consistent with a diagnosis of dementia, were eligible to participate in the study. Written informed consent to participate was obtained from those eligible residents who were considered to have capacity to consent. For those residents deemed not to have capacity to consent for themselves, written assent was obtained from a personal consultee who, based on his/her knowledge of the resident, could provide an opinion as to whether the resident would have consented to his/her care notes being reviewed. Residents with dementia whom the care home manager thought it inappropriate to approach (for example, residents in the terminal stage of the disease), or who lacked capacity to consent and for whom a consultee could not be identified, were excluded from the study [39].

Medication data for each participating resident were collected at baseline and at two further time-points using medication administration records obtained from the care home notes which detailed the medications prescribed and administered by care home staff over the four-week period which coincided with each data collection time-point. The maximum number of medications prescribed for and administered to the resident at any one time during this four-week period was utilised to determine drug use at each data collection time-point. All medications were categorised using the Anatomical Therapeutic Chemical (ATC) classification system as recommended by the World Health Organisation [40]. As per previous research examining sedative load of medications [33,34], medications taken regularly were considered, while medications used on a *pro re nata *(prn) basis were excluded. Medications were considered to be regularly taken if there was a documented regular sequence of administration. Psychotropic medications were defined as antipsychotics (ATC classification N05A), antidepressants (N06A), hypnotics (N05C) and anxiolytics (N05B). The sedative load for each resident was calculated using the sedative load model which classifies medications into four groups (Group 1: primary sedatives; Group 2: medications with sedation as a prominent side-effect or preparations with a sedating component; Group 3: medications with sedation as a potential ADE; Group 4: all other medications with no known sedative properties). Medications in groups 1 and 2 were given sedative ratings of 2 and 1 respectively, and the overall sedative load for each resident was calculated using the following formula:

$$\text{Sedative Loading }\left(\text{SL}\right)=\overset{\text{n}}{\underset{\text{k}=1}{\sum }}\text{S}{\text{R}}_{\text{k}}$$

where: n = number of drugs and SR_k _= sedative rating for drug k [29].

Data were analysed using PASW Statistics 18.0 software (SPSS Inc. Chicago, Illinois). Chi-squared (χ2) tests were used to compare categorical variables and Kruskal-Wallis tests were used to compare continuous variables. Significance was set *a priori *at p ≤ 0.05.

## Results

A total of 214 residents across the six care homes were eligible for participation in the EVIDEM-EOL study. Of these, 133 residents were recruited (62.2%). Details of the recruitment of residential care homes and residents have been previously published [39].

At baseline, medication administration records for 115 residents (86.5%) were reviewed; medication administration record sheets were unavailable for 18 residents as they were archived or otherwise unobtainable as the resident had died. The mean age of the residents was 85.8 (SD 6.8) years and 91 of these residents (79.1%) were female; the demographic profile of residents included in the study is detailed in Table 1. Between two and four general practice surgeries provided services to each care home; care homes 1, 2, 5 and 6 were each served by two general practice surgeries, while care homes 3 and 4 were each served by four general practice surgeries.

**Table 1:** Demographic profile for residential care home residents with dementia

Parameter	Care home at each time point																	
	1			2			3			4			5			6		
	BL	TP2	TP3	BL	TP2	TP3	BL	TP2	TP3	BL	TP2	TP3	BL	TP2	TP3	BL	TP2	TP3
**Number of residents (n)**	20	20	19	17	17	16	14	14	14	22	22	21	30	27	25	12	12	10
**Gender**																		
Female [no.(%)]	18 (90.0)	18 (90.0)	17 (89.5)	14 (82.4)	14 (82.4)	13 (81.3)	12 (85.7)	12 (85.7)	12 (85.7)	20 (90.9)	20 (90.9)	20 (95.2)	19 (63.3)	17 (63.0)	18 (72.0)	8 (66.7)	8 (66.7)	7 (70.0)
Male [no.(%)]	2 (10.0)	2 (10.0)	2 (10.5)	3 (17.6)	3 (17.7)	3 (18.7)	2 (14.3)	2 (14.3)	2 (14.3)	2 (9.1)	2 (9.1)	1 (4.8)	11 (36.7)	10 (37.0)	7 (28.0)	4 (33.3)	4 (33.3)	3 (30.0)
**Age**																		
Mean	87.4	87.7	88.4	87.3	88.6	87.9	89.0	89.9	90.4	84.6	85.7	85.9	83.5	83.9	83.2	83.7	84.0	84.9
Standard deviation	6.16	6.14	6.25	7.56	8.81	7.75	5.87	5.70	5.62	6.63	5.87	5.97	6.63	6.88	6.21	8.28	8.28	8.53

**BL **- baseline, **TP **- time point

At baseline, eighty-six residents (74.8%) had a recorded diagnosis of dementia in their care home notes, although 33 (38.4%) of these residents did not have a specific type of dementia recorded. Twenty-nine residents (25.2%) did not have a diagnosis of dementia in their care home notes, but were determined by care home staff as having cognitive impairment consistent with a diagnosis of dementia. None of the care homes recorded severity of dementia in their care notes but behavioural and communication difficulties arising from having dementia were recorded. According to the care home notes, a specific cognitive test, such as the Mini Mental State Examination (MMSE [41]), was administered to 23 residents (20.0%). Fifty residents (43.5%) were recorded as having 3 or more co-morbidities (obtained from the care home notes), and the mean number of long-term conditions per resident was 2.46 (SD 1.46). The Cornell Scale for Depression in Dementia [42] was applied by the care home manager, or senior care home staff, for the 91 residents for whom the required data was available. Ten residents (11.0%) scored 12 or more (indicating probable depression) and two of the care homes (3 and 4) did not identify symptoms of depression in any of their residents. Twenty-five residents (21.7%) were recorded as having a diagnosis of depression in their care home notes.

The number of residents with sedative load scores of 0 - 6 at each time-point are detailed in Table 2. Approximately one-third of residents had a sedative load score of 0 (33.0%, 26.8% and 28.5% at baseline and time-points 2 and 3 respectively), and a significant proportion of residents had a low sedative load score of 1 or 2 (54.8%, 59.0% and 57.1% at baseline and time-points 2 and 3 respectively), while 12.2% had a high sedative load (≥ 3) at baseline, increasing slightly to 14.3% at time-points 2 and 3.

**Table 2:** Sedative load for residential care home residents with dementia

	Number of residents with sedative load at each time point																				
	Care Home 1			Care Home 2			Care Home 3			Care Home 4			Care Home 5			Care Home 6			Total (%)		
Sedative Load	BL	TP 2	TP 3	BL	TP 2	TP 3	BL	TP 2	TP 3	BL	TP 2	TP 3	BL	TP 2	TP 3	BL	TP 2	TP 3	BL	TP 2	TP 3
**0**	8	6	4	3	4	7	5	5	6	6	4	5	12	10	7	4	1	1	38 (33.0)	30 (26.8)	30 (28.5)
**1**	8	7	8	11	9	7	5	4	4	5	6	5	8	9	9	4	7	5	41 (35.7)	42 (37.5)	38 (36.2)
**2**	1	3	3	1	3	1	1	2	0	9	9	9	8	5	5	2	2	4	22 (19.1)	24 (21.4)	22 (21.0)
**3**	2	3	3	1	1	1	2	1	2	2	3	2	2	2	2	2	2	0	11 (9.6)	12 (10.7)	10 (9.5)
**4**	0	0	0	0	0	0	1	2	1	0	0	0	0	1	2	0	0	0	1 (0.9)	3 (2.7)	3 (2.9)
**5**	1	1	1	0	0	0	0	0	1	0	0	0	0	0	0	0	0	0	1 (0.9)	1 (0.9)	2 (1.9)
**6**	0	0	0	1	0	0	0	0	0	0	0	0	0	0	0	0	0	0	1 (0.9)	0 (0)	0 (0)
**Total**	20	20	19	17	17	16	14	14	14	22	22	21	30	27	25	12	12	10	115 (100.0)	112 (100.0)	105 (100.0)

**BL **- baseline, **TP **- time point

Use of regular medications is outlined in Table 3. At baseline, the mean number of regular medications prescribed for and administered to each resident ranged from 4.36 (SD 2.11) in care home 4 to 7.41 (SD 4.83) in care home 2. Prevalence of polypharmacy, defined by the National Service Framework for Older People [43] as the prescription of four or more medications, varied within the study population; residents in care home 6 had significantly lower levels of polypharmacy than residents in care home 5. Approximately two-thirds of residents (n = 77, 66.9%) regularly used one or more psychotropic medication(s) [an antipsychotic, hypnotic, anxiolytic or antidepressant].

**Table 3:** Use of regular medications, including sedative medications, by residential care home residents with dementia

Parameter	Care home at each time point																		**p - Value**		
	1			2			3			4			5			6					
	BL	TP2	TP3	BL	TP2	TP3	BL	TP2	TP3	BL	TP2	TP3	BL	TP2	TP3	BL	TP2	TP3	BL	TP2	TP3
**Number of residents (n)**	20	20	19	17	17	16	14	14	14	22	22	21	30	27	25	12	12	10			
**Number of regular medications**																					
Mean	6.70	5.10	5.11	7.41	5.88	6.00	5.79	5.57	5.57	4.36	4.41	4.33	6.17	6.74	6.88	5.17	5.75	5.90	0.07	0.13	0.10
Range	1-10	1-8	1-8	2-18	2-11	2-11	1-10	0-13	0-13	0-8	0-9	1-9	0-10	0-13	0-13	0-12	0-12	1-12			
Standard deviation	3.18	2.40	2.47	4.83	3.06	3.12	3.17	3.28	3.28	2.11	2.32	2.35	2.51	3.58	3.59	3.86	3.14	3.51			
**Polypharmacy (>4 medications) [no.(%)]**	16 (80.0)	18 (90.0)	14 (73.6)	15 (88.2)	14 (82.3)	12 (75.0)	9 (64.2)	5 (35.7)	10 (74.1)	14 (63.6)	16 (72.7)	11 (52.4)	25 (83.3)	20 (74.0)	23 (92.0)	7 (58.3)	5 (41.7)	9 (90.0)	0.02	0.19	0.08
**Sedative Load**																					
Mean	1.05	1.35	1.47	1.29	1.06	0.75	1.21	1.36	1.36	1.32	1.50	1.38	1.03	1.07	1.32	1.08	1.42	1.30	0.84	0.59	0.39
Range	0-5	0-5	0-5	0-6	0-3	0-3	0-4	0-4	0-5	0-3	0-3	0-3	0-3	0-4	0-4	0-3	0-3	0-2			
Standard deviation	1.32	1.35	1.31	1.40	0.83	0.86	1.31	1.45	1.69	0.99	0.96	0.97	0.98	1.11	1.22	1.12	0.9	0.68			
**Antipsychotics [no.(%)]**	2 (10.0)	4 (20.0)	5 (26.3)	7 (41.2)	6 (35.3)	4 (25.0)	2 (14.3)	1 (7.1)	1 (7.1)	7 (32.0)	6 (27.3)	5 (23.8)	3 (10.0)	3 (11.1)	4 (16.0)	2 (16.8)	1 (8.3)	0	0.02	0.17	0.04
**Antidepressants [no.(%)]**	11 (55.0)	12 (60.0)	11 (57.9)	9 (52.9)	6 (35.3)	4 (25.0)	5 (35.7)	8 (57.1)	9 (64.3)	15 (68.2)	14 (63.6)	13 (61.9)	10 (33.3)	11 (40.7)	11 (44.0)	4 (33.3)	6 (50.0)	6 (60.0)	0.29	0.41	0.35
**Hypnotics and anxiolytics [no.(%)]**	1 (5.0)	1 (5.0)	1 (5.3)	1 (5.9)	0	0	3 (21.4)	3 (21.4)	2 (14.3)	2 (9.1)	2 (9.1)	2 (9.5)	4 (13.3)	5 (18.5)	5 (20.0)	2 (16.7)	3 (25.0)	2 (20.0)	0.06	0.14	0.23

**BL **- baseline, **TP **- time point

Data relating to individual drug classes showed variability across the study population, as presented in Table 4. The use of antipsychotic medications ranged from 10.0% to 41.2% across care homes, with care home 2 found to have a considerably higher level of antipsychotic prescribing than the other homes. The most commonly used atypical antipsychotic was quetiapine (n = 14, 12.2%) as shown in Table 5. All residents taking antipsychotics in care home 1 were prescribed conventional antipsychotics (Tables 4 and 5).

**Table 4:** Use of classes of psychotropic medications by residential care home residents with dementia

Drug Class	Care home data at each time point																			
	1			2			3			4			5			6				
	BL	TP 2	TP 3	BL	TP 2	TP 3	BL	TP 2	TP 3	BL	TP 2	TP 3	BL	TP 2	TP 3	BL	TP 2	TP 3		
**Antipsychotics**																				
Conventional [no.(%)]	2 (10.0)	4 (20.0)	5 (26.3)	0	0	0	1 (7.1)	1 (7.1)	1 (7.1)	1 (4.6)	1 (4.6)	1 (4.8)	1 (3.3)	1 (3.7)	2 (8.0)	1 (8.3)	1 (8.3)	0		
Atypical [no.(%)]	0	0	0	7 (41.2)	6 (35.3)	4 (25.0)	1 (7.1)	0	0	6 (27.3)	5 (22.7)	4 (19.0)	2 (6.7)	2 (7.4)	2 (8.0)	1 (8.3)	0	0		
**Antidepressants**																				
TCAs [no.(%)]	0	1 (5.0)	0	3 (17.7)	1 (5.9)	1 (6.3)	1 (7.1)	1 (7.1)	1 (7.1)	1 (4.6)	0	0	0	0	1 (4.0)	0	0	0		
SSRIs [no.(%)]	11 (55.0)	11 (55.0)	10 (52.6)	6 (35.3)	5 (29.4)	3 (18.8)	4 (28.6)	4 (28.6)	5 (35.7)	2 (9.1)	2 (9.1)	2 (9.5)	7 (23.3)	9 (33.3)	7 (28.0)	3 (25.0)	5 (41.7)	5 (50.0)		
Other antidepressants [no.(%)]	0	0	1 (5.3)	0	0	1 (6.3)	0	3 (21.4)	3 (21.4)	12 (54.6)	12 (54.6)	11 (52.4)	3 (10.0)	2 (7.4)	3 (12.0)	1 (8.3)	1 (8.3)	1 (10.0)		
**Hypnotics [no.(%)]**	1 (5.0)	1 (5.0)	1 (5.3)	0	0	0	2 (14.3)	2 (14.3)	1 (7.1)	1 (4.6)	1 (4.6)	1 (4.8)	2 (6.7)	2 (7.4)	2 (8.0)	2 (16.7)	3 (25.0)	2 (20.0)		
**Anxiolytics [no.(%)]**	0	0	0	1 (5.9)	0	0	1 (7.1)	1 (7.1)	1 (7.1)	1 (4.6)	1 (4.6)	1 (4.8)	2 (6.7)	3 (11.1)	3 (12.0)	0	0	0		

**BL **- baseline, **TP **- time point

**TCA **- tricyclic antidepressant; **SSRI **- selective serotonin re-uptake inhibitor

**Table 5:** Prevalence of the four most commonly used primary sedatives and drugs with sedation as a prominent adverse effect/containing a sedating component among residential care home residents with dementia

Variable	Drug Name	All residents	Care Home					
			1	2	3	4	5	6
		n = 115	n = 20	n = 17	n = 14	n = 22	n = 30	n = 12
**Baseline**								
**Primary Sedatives [no.(%)]**	Promazine	5 (4.4)	2 (10.0)	0	1 (7.1)	1 (4.5)	1 (3.3)	0
	Temazepam	4 (3.5)	0	0	1 (7.1)	1 (4.5)	0	1 (8.3)
	Lorazepam	3 (2.6)	0	1 (5.9)	1 (7.1)	1 (4.5)	0	0
	Amitriptyline	3 (2.6)	2 (10.0)	1 (5.9)	0	0	0	0
**Drugs with sedation as a prominent adverse effect/**	Citalopram	18 (15.7)	6 (30.0)	2 (11.8)	1 (7.1)	1 (4.5)	5 (16.7)	3 (25.0)
**containing a sedating component [no.(%)]**	Quetiapine	14 (12.2)	0	6 (35.3)	1 (7.1)	5 (22.7)	1 (3.3)	1 (8.3)
	Trazodone	12 (10.4)	0	0	0	11 (50.0)	1 (3.3)	0
	Fluoxetine	8 (7.0)	3 (15.0)	3 (17.6)	1 (7.1)	1 (4.5)	0	0

**Timepoint 2**		n = 112	n = 20	n = 17	n = 14	n = 22	n = 27	n = 12
**Primary Sedatives [no.(%)]**	Promazine	5 (4.4)	2 (10.0)	0	1 (7.1)	1 (4.5)	1 (3.7)	0
	Temazepam	4 (3.6)	0	0	1 (7.1)	1 (4.5)	0	2 (16.7)
	Zopiclone	4 (3.6)	1 (5.0)	0	0	0	2(7.4)	1 (8.3)
	Lorazepam	3 (2.7)	0	0	1 (7.1)	1(4.5)	1 (3.7)	0
**Drugs with sedation as a prominent adverse effect/**	Citalopram	20 (17.9)	6 (30.0)	1 (5.9)	1 (7.1)	1 (4.5)	6 (22.2)	5 (41.7)
**containing a sedating component [no.(%)]**	Trazodone	15(13.4)	0	0	3(21.4)	11 (50.0)	1 (3.7)	0
	Quetiapine	11 (9.8)	0	5 (29.4)	0	5 (22.7)	1 (3.7)	0
	Fluoxetine	9 (8.0)	3 (15.0)	3 (17.6)	1 (7.1)	1 (4.5)	1 (3.7)	0

**Timepoint 3**		n = 105	n = 19	n = 16	n = 14	n = 21	n = 25	n = 10
**Primary Sedatives [no.(%)]**	Promazine	7 (6.7)	2 (10.0)	0	1 (7.1)	1 (4.8)	2 (8.0)	0
	Temazepam	3 (2.9)	0	0	1 (7.1)	1 (4.8)	0	1 (8.3)
	Zopiclone	3 (2.9)	0	0	0	0	2 (8.0)	0
	Lorazepam	3 (2.9)	2 (10.0)	0	1 (7.1)	1 (4.8)	1 (4.0)	0
**Drugs with sedation as a prominent adverse effect/**	Citalopram	18 (15.7)	5 (26.0)	0	2 (14.3)	1 (4.8)	5 (20.7)	5 (50.0)
**containing a sedating component [no.(%)]**	Trazodone	15 (14.3)	0	0	3 (21.4)	10 (47.6)	1 (4.0)	1 (10.0)
	Quetiapine	9 (8.6)	0	4 (25.0)	0	4 (19.0)	1 (4.0)	0
	Fluoxetine	8 (7.6)	3 (15.8)	2 (12.5)	1 (7.1)	1 (4.8)	1 (4.0)	0

**BL **- baseline, **TP **- time point

Regular use of antidepressant medication ranged from 33.3% in care home 5 to 68.2% in care home 4. Selective serotonin re-uptake inhibitors (SSRIs) were the most widely used class of antidepressants across all care homes with the exception of care home 4, where trazodone prescribing predominated. This finding is presented in Table 4, in which trazodone is categorised as 'other antidepressants'. Twenty-one of the 22 residents in care home 4 were assessed at baseline using the Cornell Scale for Depression in Dementia [42], and all of these residents were classified as unlikely to be suffering from depression (a Cornell score of less than 12), indicating that trazodone may have been prescribed for treatment of behavioural symptoms such as agitation. However, when assessed using criteria for agitated/not agitated status adapted from the Cohen-Mansfield Agitation Inventory [44], these residents did not show significantly higher scores for agitation than residents in the other care homes (χ2 test; p > 0.05). This indicated that trazodone may have instead been used for treatment of insomnia. Trazodone was only prescribed for one other resident in care home 5, and was not prescribed for any of the ten residents in the study who were determined to be suffering from probable depression (a Cornell score equal to or more than 12). Citalopram was the most frequently prescribed SSRI in the care homes (n = 18, 15.7%; table 5).

Regular administration of hypnotics and anxiolytics was low, ranging from 5.0% in care home 1 to 21.4% in care home 3, while the most frequently used hypnotic and anxiolytic medications were temazepam (3.5%) and lorazepam (2.6%) respectively (Table 5). Hypnotics were not used by residents in care home 2, while residents in care homes 1 and 6 did not use anxiolytics (Table 4).

At time-points 2 and 3, the study population declined to 112 and to 105 participants respectively due to drop-out and death. The demographic data, prevalence of psychotropic drug use, mean number of regular medications and prevalence of polypharmacy are presented in Tables 1 and 2 and are similar to those presented at baseline, although at time-point 3, there was a significant difference in mean age of the residents in care homes 3 (90.4 years) and 5 (83.2 years) [Kruskal-Wallis test, p = 0.02]. At time-points 2 and 3, as at baseline, the most commonly used atypical antipsychotic was quetiapine (Table 5). At time-point 2, conventional antipsychotics were not used by residents in care home 2 and atypical antipsychotics were not used by residents in care homes 1, 3 and 6. In care homes 4 and 5 both conventional and atypical antipsychotics were used, with atypical antipsychotics constituting the majority of antipsychotic use. SSRIs were the most commonly used antidepressants, with use ranging from 9.1% in care home 4 to 55.0% in care home 1 (Table 4). Table 5 shows that the most frequently administered hypnotics were temazepam and zopiclone (each at 3.6%), while lorazepam was the most regularly used anxiolytic medication (2.7%).

At time-point 3, variation in the use of antipsychotics was apparent within the population (Table 3, χ2, p = 0.04). Moreover, antipsychotics were not administered on a regular basis for any resident in care home 6. Conventional antipsychotics were used in care homes 1, 3, 4 and 5 but not in care homes 2 and 6. Promazine was the most frequently administered conventional antipsychotic (6.7%). Use of antidepressants ranged from 25.0% in care home 2 to 64.3% in care home 3 (Table 3). As at baseline and time-point 2, SSRIs were the most frequently used antidepressants ranging from 9.5% in care home 4 to 52.6% in care home 1 (Table 4). Tricylic antidepressants were not used in care homes 1, 4 or 6. In care home 2, hypnotics and anxiolytics were not administered (Table 5). Furthermore, it is important to note that throughout the duration of this study, use of medications which are recognised as having prominent sedative adverse effects and/or containing sedative components outweighed the regular use of primary sedatives (Table 5).

## Discussion

This study analysed the sedative load of medications regularly taken by older people with dementia living in six residential care homes in England, using the sedative load model [35,36]. This model has previously been used to examine the sedative load for residents with and without dementia in long-term care wards [33,34]. However, prior to the current study, it has not been used in settings where there is no on-site clinician.

The study population was more frequently administered medications with sedation as a prominent adverse effect or preparations with a sedating component rather than primary sedatives. Psychotropic medications were used on a regular basis by approximately two-thirds of residents in this study. This complements studies in nursing homes which have reported that 67% to 78% of patients with dementia were prescribed at least one psychotropic medication [21,45,46].

Despite the variation in prescribing, sedative load scores were similar for residents across care homes at each time-point. These scores were lower than have been previously reported in residents of long-term care facilities and home-dwelling older people [34,35]. However, the findings of this study support a US study of long-term care facilities, in which residents with dementia were found to have a low sedative load [26].

Whilst some studies have reported that patients with dementia are frequently prescribed antipsychotics and hypnotics [20,47], this study found that across all care homes, antidepressants were more frequently used by residents (49.8%), with antipsychotics (19.0%) and hypnotics and anxiolytics (11.7%) less routinely administered. However, this difference may in part by explained by the fact that in this study medications used on a *pro re nata *(prn) basis were excluded, whilst previous studies [20,47] included these medications.

The most commonly used antidepressants in this study were SSRIs with citalopram being the most regularly used. This supports the findings from previous studies [34,46]. Fluoxetine was administered to 15% of the residents throughout the study period, despite its long half- life which may make it inappropriate for use in older people [48]. In care home 4, SSRIs were less commonly used and trazodone use predominated, which may be explained by an association between this care home and a memory clinic. Across the study period, regular use of hypnotics (ranging from 0% to 25.0%) was similar to previous studies, with regular use of anxiolytics lower than previously reported [21,34].

At 19.0%, the prevalence of antipsychotic use among residents with dementia in this study was lower than previously reported in nursing home residents [21,34,46]. Overall, atypical antipsychotics were more commonly used than conventional antipsychotics, reflecting prescribing trends and the evidence that these medications are superior in controlling the behavioural and psychological symptoms of dementia [49], contribute less to the sedative load score and are associated with fewer extrapyramidal side-effects. However, care home 1 was a notable exception; throughout the study period, all residents taking antipsychotics used conventional antipsychotics. In care home 2 at baseline, antipsychotic use was significantly higher than in the other care homes. The resident characteristics may have contributed to this finding, since a high percentage of residents had three or more medical conditions recorded in their notes.

Previous studies have reported that the most frequently prescribed psychotropic medications for patients with dementia were antipsychotics [34,46]; however in this study antidepressants were predominantly used. This finding merits further investigation, using a larger population.

The underpinning knowledge for prescribing specific psychotropic medications and their impact on the symptoms and quality of life experienced by patients with dementia should be the focus of subsequent studies. Formal trials of psychotropic drugs with low sedative loading in care home residents with dementia should also be undertaken. Further research is also required into non-pharmacological alternatives to psychotropic medication, building upon the work conducted to date [50-63].

This study also highlights the lack of standardised diagnosis and staging of dementia in residential care home residents. One-quarter of the study participants did not have a diagnosis of dementia in their care home notes, but were determined by care home staff as having cognitive impairment consistent with a diagnosis of dementia. Furthermore, none of the care homes recorded severity of dementia in their care notes. There is a need for accurate and standardised diagnosis and staging of dementia in residential care homes to ensure that residents receive appropriate and timely care, despite the potential challenges such an approach may bring.

There are a number of methodological strengths of this study. The longitudinal design allowed examination of the pattern of medication use over the study period, while the use of an objective method to calculate the cumulative sedative effect of multiple medications eliminated subjective bias. Data collection directly from residents' medication administration records rather than from computerised data records facilitated the identification of medicines which were regularly administered rather than those which were dispensed but not actually taken by the patient.

It is also important to consider the methodological limitations of this study. Potential participants were identified either through a documented diagnosis of dementia within residents' care home notes or by senior care home staff as having cognitive impairment consistent with a diagnosis of dementia. The sedative load model presents inherent drawbacks including the exclusion of Group 3 drugs, which have the potential to cause sedation [36]. Furthermore, this model does not consider doses and frequencies of medications [34]. As with previous studies [33,34], medications taken 'when required' (prn) were excluded from the analysis. These medications could further contribute to an individual's sedative load.

## Conclusions

To optimise drug therapy for patients with dementia there is a need to take into consideration how individual drugs interact and contribute to the total sedative load score of the medications prescribed. In this study, residents with dementia were more frequently administered drugs which had sedation as a prominent adverse effect and/or contained a sedating component rather than primary sedatives.

Antidepressants, predominantly SSRIs, were most frequently administered, while levels of use of hypnotics and anxiolytics were lower. Sedative load scores were found to be similar throughout the study period for residents with dementia in each of the six care homes, and were lower than previously reported in other long-term care settings. This study focused on older people with dementia in care homes which have no on-site nursing or medical provision. It demonstrates the necessity for further research with this population and the need to develop interventions which support ongoing review and discussion between health service and care home providers. These interventions must recognise the complexities of working across systems of care with a workforce unqualified in medication review.

## Competing interests

The authors declare that they have no competing interests.

## Authors' contributions

CP drafted the manuscript and participated in the analysis and interpretation of the data. JEH participated in the analysis and interpretation of the data. NB, EM, IM and ES supported the data collection and analysis of the findings and commented on drafts of the manuscript. SA supported data analysis and final checks on data. CG designed the study and is the lead investigator, contributed sections of the manuscript and commented on all drafts. All authors read and approved the final manuscript.

## Pre-publication history

The pre-publication history for this paper can be accessed here:

http://www.biomedcentral.com/1471-2318/11/56/prepub
